# The diagnostic and therapeutic value of endoscopic irrigation for cecal suppurative diverticulitis: A case report

**DOI:** 10.1097/MD.0000000000049551

**Published:** 2026-07-10

**Authors:** Chun Gao, Jiu-Cong Zhang

**Affiliations:** a Department of Gastroenterology, The 940 Hospital of Joint Logistic Support Force of PLA, Lanzhou, Gansu, China.

**Keywords:** cecal diverticulitis, diagnosis, endoscopic examination, endoscopic therapy

## Abstract

**Rationale:**

Cecal diverticulitis closely mimics acute appendicitis in clinical presentation, leading to frequent misdiagnosis. Traditional mistaken appendectomy is not only ineffective but may also delay proper treatment, risking serious complications such as perforation. Abdominal computed tomography plays a key role in differential diagnosis. This report explores the value of endoscopic irrigation as a novel diagnostic and therapeutic approach.

**Patient concerns:**

A patient presented with symptoms strongly suggestive of acute appendicitis, prompting further diagnostic workup to rule out alternative causes.

**Diagnoses:**

Preoperative computed tomography suggested suppurative cecal diverticulitis, which was subsequently confirmed during colonoscopy.

**Interventions:**

Targeted endoscopic irrigation and drainage were performed directly into the confined diverticular abscess cavity, with real-time visualization allowing extraction of impacted fecaliths and purulent debris.

**Outcomes:**

Following endoscopic irrigation, the patient’s symptoms resolved rapidly, resulting in significant improvement and discharge. At 4- and 6-month follow-ups, there was no recurrence of diverticulitis or related complications.

**Lessons:**

For carefully selected patients, endoscopic irrigation demonstrates dual diagnostic and therapeutic value in suppurative cecal diverticulitis. This minimally invasive approach effectively avoids unnecessary surgery, alleviates symptoms, and shows promising short- and mid-term outcomes. Further studies are needed to validate its broader applicability, and patient selection remains critical.

## 1. Introduction

Diverticula of the digestive tract are round or oval sac-like bulges protruding from the lumen of the digestive tract and can be found throughout the digestive tract, with the highest incidence in the colon, where diverticula are often multiple.^[1]^ Right-sided diverticulitis occurs in only 1.5% of cases, mainly in the anterior portion of the cecum, proximal to the ileocecal valve (80%).^[2]^ Acute colonic diverticulitis occurs in approximately 10% to 25% of colonic diverticula,^[3]^ and cecal diverticulitis is often misdiagnosed and underdiagnosed due to the lack of specific clinical manifestations and means of confirming the diagnosis. Cecal diverticulitis can be complicated by abdominal abscess and perforation, and conservative treatment alone has limited efficacy. In recent years, endoscopic techniques have demonstrated unique value in the minimally invasive management of inflammatory diseases and abscesses of the digestive tract. For instance, endoscopic ultrasound (EUS)-guided drainage of colonic abscesses has achieved favorable outcomes in select complex cases. Similarly, endoscopic retrograde appendicitis therapy (ERAT), which involves endoscopic irrigation, drainage, and fecalith removal, has established an organ-preserving treatment paradigm for appendiceal lumenal inflammation. These advances suggest that direct endoscopic intervention targeting the diverticular cavity – a confined space – may serve as a valuable complement to conventional treatment. Compared with percutaneous drainage, endoscopic drainage avoids the need for an indwelling external drain and its associated complications, while enabling simultaneous diagnosis and treatment. This approach offers particular advantages for lesions located deep within the abdominal cavity or in anatomically complex regions. This article reports a case of purulent diverticulitis of the cecum misdiagnosed as acute appendicitis treated with endoscopic irrigation, which achieved good results. It is reported as follows:

## 2. Case presentation

Female, 42 years old, admitted to the hospital due to “Migrating pain to the right lower quadrant for more than a day.” The pain started below the xiphoid process and gradually spread and fixed in the lower right abdomen. It intensified intermittently and was persistent, with severe and unbearable pain. There was no fever, nausea, vomiting, lower back pain, perineal pain, or radiation pain from external genitalia. Abdominal palpation revealed obvious tenderness at the McBurney’s point in the right lower abdomen, with rebound tenderness and no significant muscle tension. Upon admission, relevant laboratory tests were completed, and the blood routine showed a white blood cell count of 16.2 × 10^9^/L and a neutrophil count of 13.50 × 10^9^/L, infection detection indicators: procalcitonin: 0.079 ng/mL, IL-6: 57.7 pg/mL. Abdominal ultrasound revealed a slightly enlarged coronal hypoechoic structure with a blind end in the appendix area of the lower right abdomen, with a diameter of approximately 0.63cm. The echo inside the tube was reduced, and no abnormal echo area was detected around it, suggesting chronic appendicitis. Based on medical history, clinical manifestations, and the aforementioned tests, the diagnosis upon admission was acute exacerbation of chronic appendicitis.

**Figure 1.**
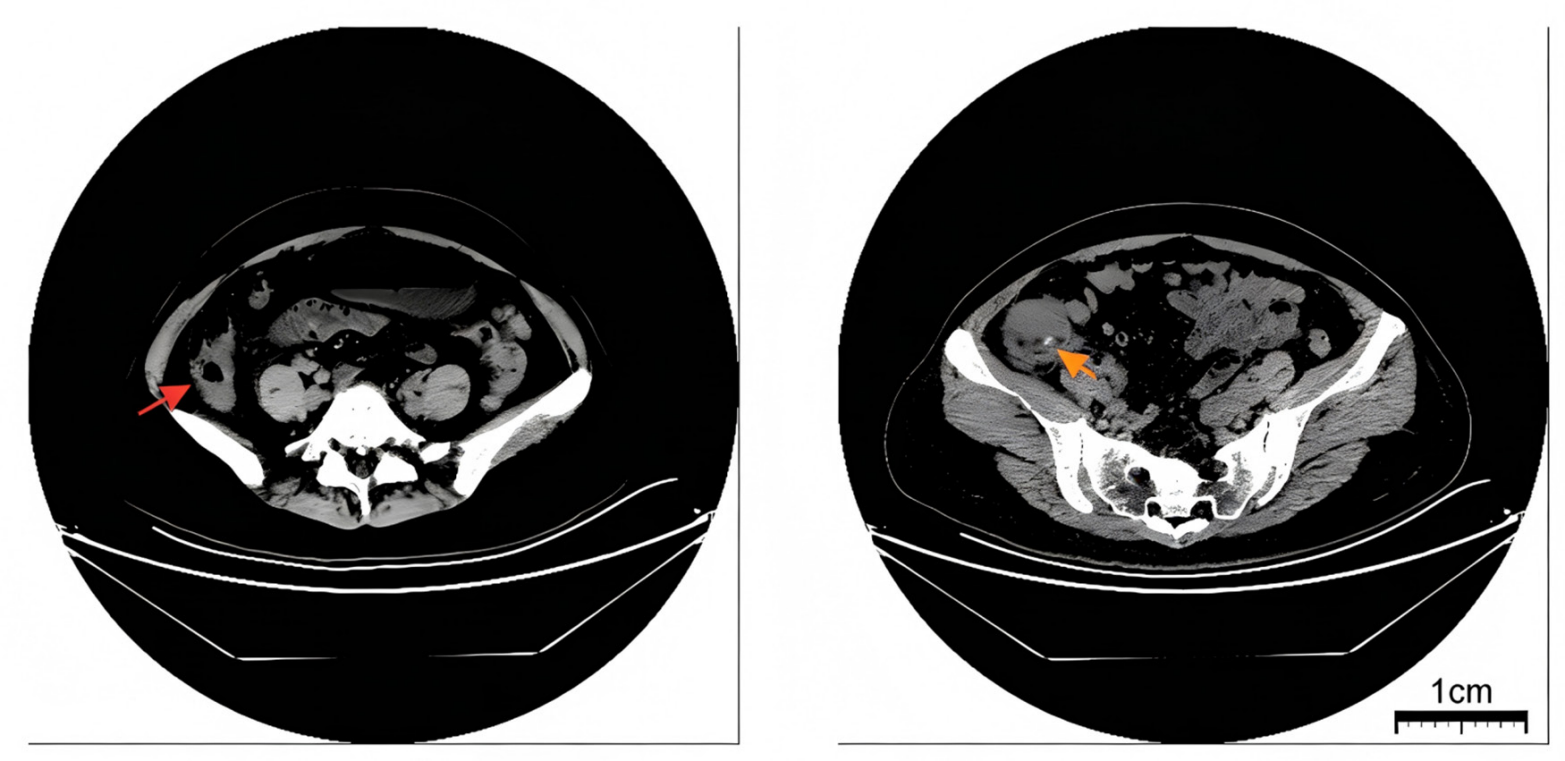
CT imagings (The red arrow indicates diverticulum, and the orange arrow indicates fecal stones). CT = computed tomography.

On the night of admission, the patient’s temperature rose to a maximum of 38.6 °C, and she was given comprehensive treatment including fasting, fluid replacement, cefoperazone sodium and sulbactam sodium for anti-infection. On the third day of admission, a follow-up blood routine showed a white blood cell count of 9.39 × 10^9^/L and a neutrophil count of 7.79 × 10^9^/L, Infection detection indicators: procalcitonin: 0.098 ng/mL, IL-6: 9.0 pg/mL. Contrast-enhanced abdominal computed tomography (CT) revealed a localized sac-like hyperdense protrusion arising from the posterior wall of the cecum, measuring approximately 1.2 cm in diameter. The wall of the sac was irregular and thickened, with surrounding pericolic fat stranding and multiple mildly enlarged lymph nodes. A fecalith was observed within the diverticulum. These findings were consistent with cecal diverticulitis. The possibility of a variant appendix with concurrent appendicitis could not be entirely excluded (Fig. 1).

**Figure 2.**
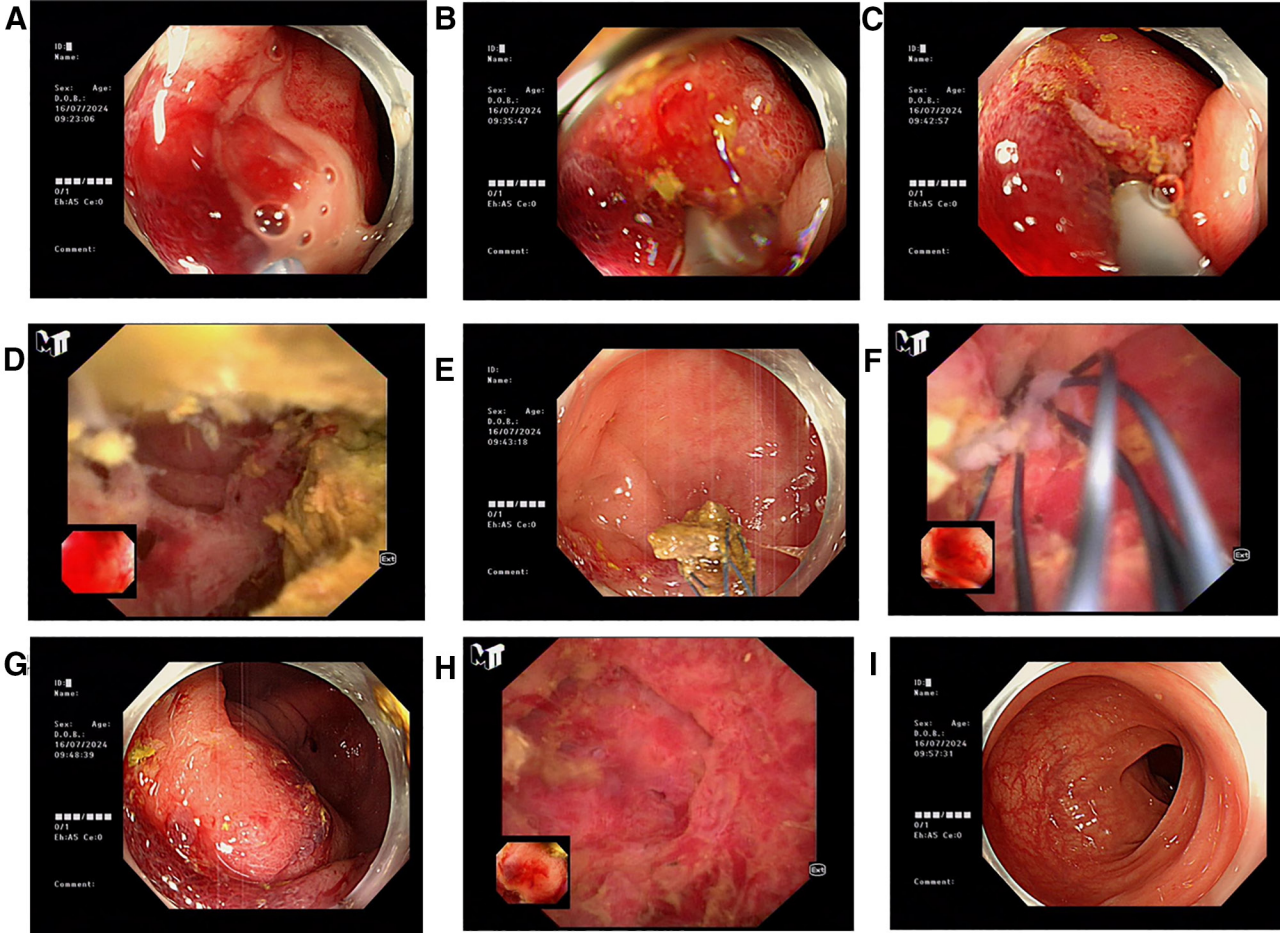
Endoscopic view and irrigation process (A) purulent discharge and surrounding mucosal congestion and edema were seen in the diverticulum; (B, C) crumbly fecal matter in the diverticulum and edema of the surrounding mucosa; (D, F) visible yellow fecal stone embedded within the diverticulum, repeated lithotripsy and stone removal using a mesh basket; (G, H) endoscopic irrigation and treatment until there is no pus outflow; and (I) retreat endoscopy.

On the 5th day of admission, the patient’s temperature have returned to normal, and they were fasted and given treatment such as fluid replacement, anti-infection, acid suppression, and nutritional support. After treatment, the patient’s condition have stabilized. Then, the patient have underwent strict bowel preparation. The first dose of sodium phosphate powder (21.6 g per packet) was dissolved in 800 ml of water and administered at 05:00, to be consumed within 30 minutes; the second dose was similarly administered at 10:00. After completing both doses, a small amount of pure water was taken orally based on the patient’s bowel cleansing status. After obtaining informed consent, we have performed endoscopic irrigation using an Olympus 290 colonoscope. The patient was placed in a left lateral position and the endoscope was inserted into the cecum near the ileocecal valve. Mucosal congestion and edema were observed, with white purulent discharge covering the surface. A contrast catheter was used to enter the diverticulum for flushing, and a large amount of white purulent discharge was seen. The endoscope was replaced to enter the diverticulum, where the mucosa was congested and eroded, and yellow fecal stones were visible embedded in the diverticulum. A mesh basket was used to repeatedly crush, remove, and flush all fecal stones (Fig. 2). The specific operation process is as follows.

Preoperative bowel preparation was performed according to standard colonoscopy requirements. A colonoscope equipped with a transparent cap was inserted to the cecum. A single-operator cholangioscope (eyeMAX; Micro-Tech Co., Ltd., Nanjing, China; instrument channel diameter > 1.0 mm) was introduced through the working channel and advanced into the diverticular cavity under direct visualization. Normal saline was intermittently irrigated (total volume approximately 500 mL) while suction was applied to evacuate purulent material; irrigation continued until the return fluid was clear. A retrieval basket was used when fecaliths were encountered. No mucosal ablation, stent placement, or clip closure was performed in this case. Tissue biopsy and microbiologic cultures were not obtained. The patient’s condition remained stable after surgery and was discharged on the 4th day.

**Figure 3.**
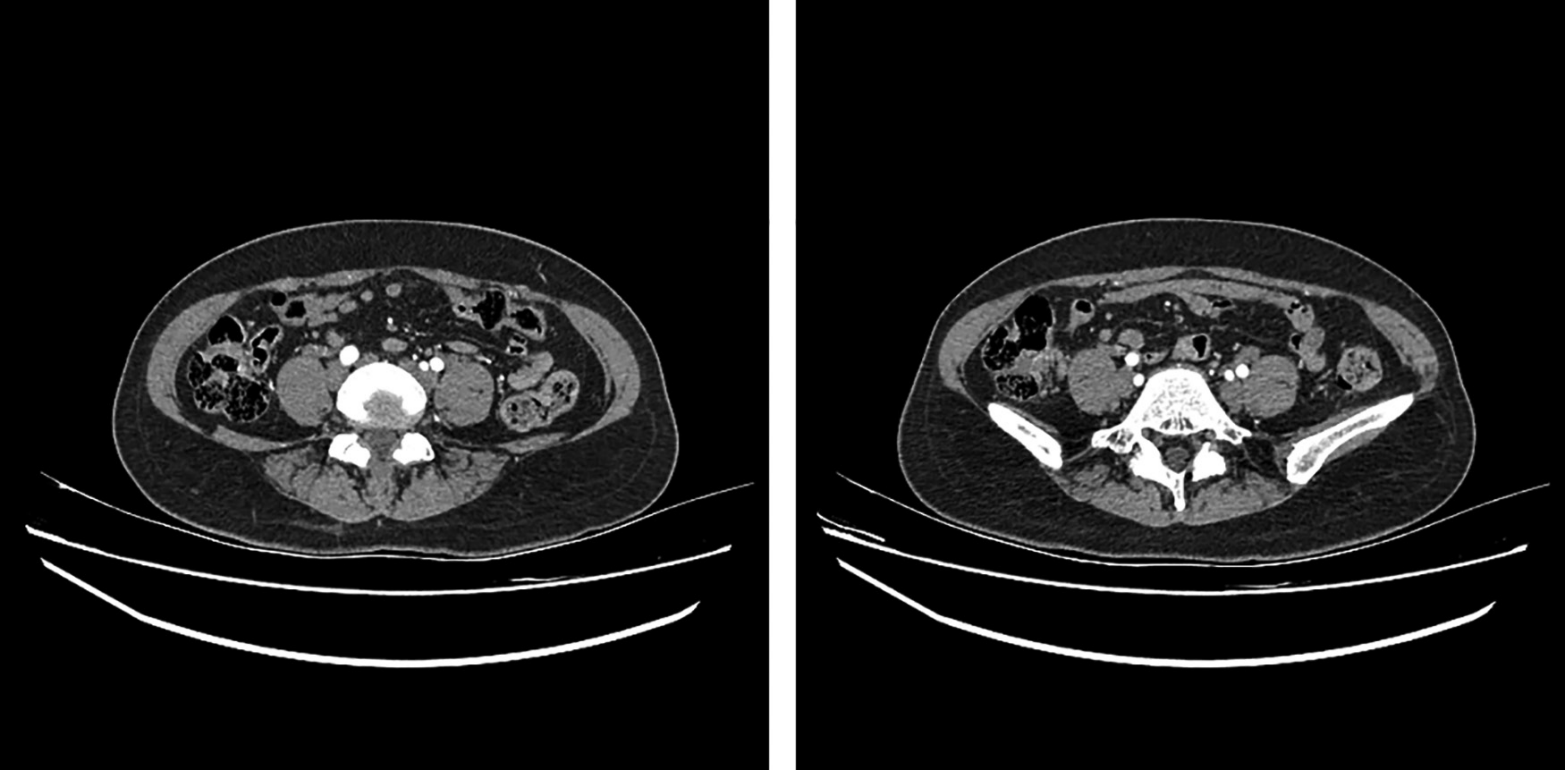
Abdominal CT scan. CT = computed tomography.

**Figure 4.**
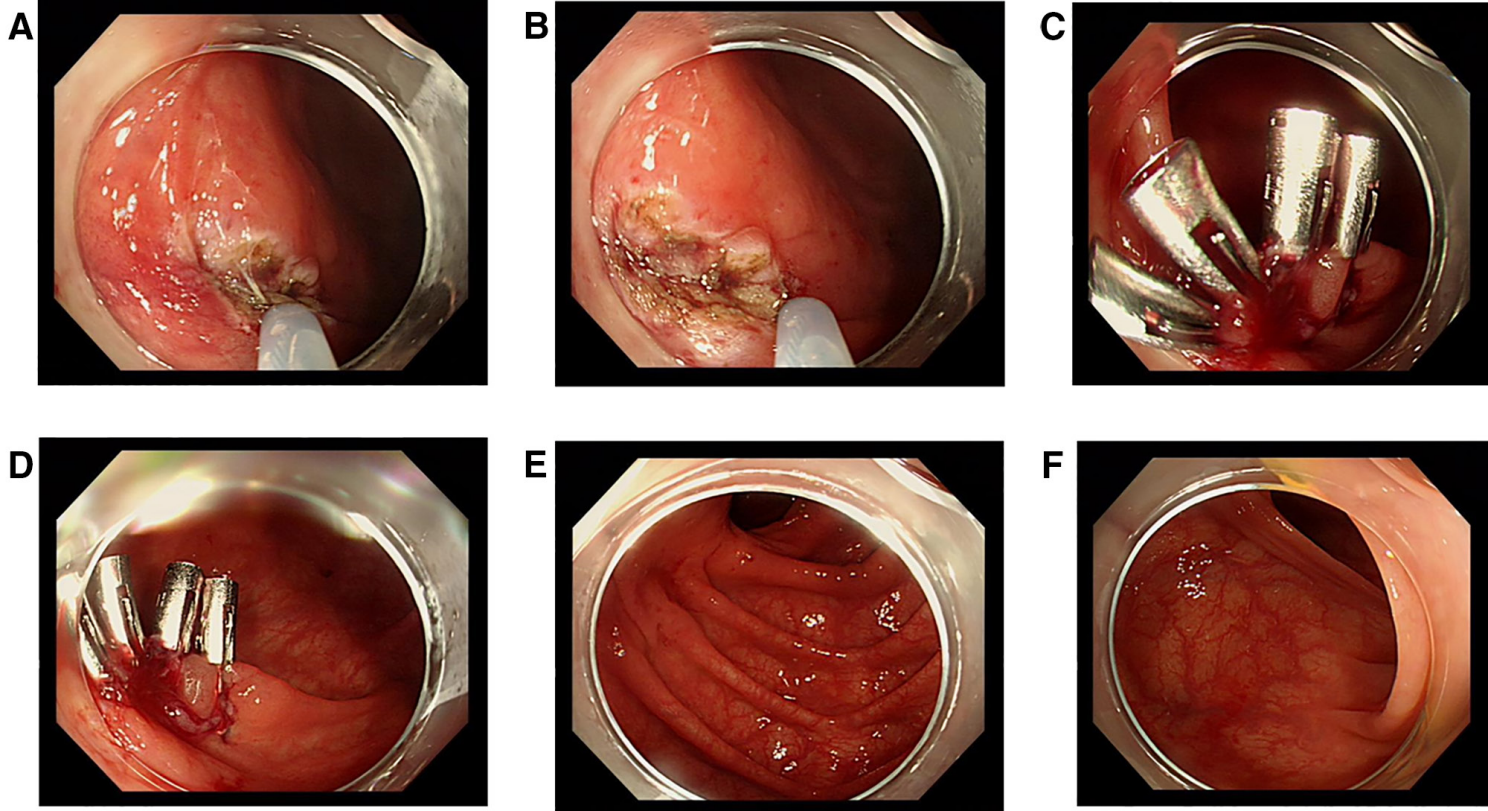
Electronic colonoscopy examination (A, B) granulation tissue hyperplasia can be seen at the diverticulum opening; (C, D) 4 harmonious clamps are used to clamp the diverticulum opening; (E, F) Smooth mucosa of the colon and rectum.

Two months after surgery, the patient’s abdominal CT scan has showed no significant thickening of the cecal wall and clear fat gaps around it (Fig. 3). Electronic colonoscopy prompt: A diverticulum opening can be seen below the ileocecal valve, and granulation tissue hyperplasia can be seen at the diverticulum opening. After repeated flushing with physiological saline, a snare is used to electrocoagulate and destroy the mucosa around the diverticulum opening. Then, 4 harmonious clamps are used to clamp the diverticulum opening, and the remaining colorectal mucosa is smooth (Fig. 4). Follow-up at 4 and 6 months after surgery showed no recurrence in the patient.

## 3. Discussion

Colonic diverticulitis represents a common colorectal emergency in Western nations, though its clinical and anatomical presentation exhibits significant geographic variation.^[4]^ While left-sided diverticulitis predominates in Western populations, right-sided diverticulitis is more frequently encountered in Asian countries, where it can account for 70% to 90% of cases.^[5]^ Pathophysiologically, right-sided diverticula are often considered congenital “true diverticula,” involving all layers of the colonic wall, as opposed to the acquired “pseudo-diverticula” more common in the sigmoid colon. The inflammation typically initiates when a fecalith obstructs the narrow neck of a diverticulum. This obstruction leads to bacterial overgrowth, impaired microvascular perfusion, and ultimately, transmural inflammation, perforation, and abscess formation.^[6-8]^ Emerging evidence also suggests potential roles for genetic susceptibility and alterations in the gut microbiome in the pathogenesis of diverticulitis, though these factors are not yet fully elucidated.^[9,10]^

The management of acute diverticulitis with abscess formation spans a conservative-to-invasive spectrum. Uncomplicated cases are often managed with bowel rest and antibiotics.^[4]^ Percutaneous drainage (PCD) combined with antibiotics achieves 70% to 90% success but requires indwelling catheter care and carries risks of tube dislodgment and tract infection.^[5,11]^ Laparoscopic surgery, while less invasive than open surgery, still carries risks of anastomotic leak and adhesions.^[12]^ Our endoscopic intervention offers distinct advantages: direct visualization of the inflamed diverticulum, targeted irrigation, and physiologic internal drainage via the native orifice, eliminating the burden of an external drain.^[11]^ This approach shares the principle of endoscopic retrograde appendicitis therapy (ERAT) in achieving organ-preserving drainage. Potential risks include perforation, bleeding, transient bacteremia, and incomplete drainage, which must be weighed against the benefits of avoiding surgery. This approach was feasible due to a small (1.2 cm), unilocular abscess with visible luminal communication, no peritonitis or free air, and anatomic accessibility. Suitable candidates should have an abscess ≤3 cm with luminal communication, no generalized peritonitis, and accessible anatomy; the procedure should be performed by an experienced endoscopist. Further studies are needed to validate these criteria.

To our knowledge, the use of endoscopic irrigation as a primary therapeutic intervention for suppurative cecal diverticulitis remains sparsely documented, making this case a valuable contribution to the literature. The successful outcome underscores that in carefully selected patients – characterized by a single, accessible, and well-contained abscess – this technique can serve as both a diagnostic confirmatory and a definitive therapeutic procedure. It can potentially prevent unnecessary appendectomies, which are a common pitfall due to the similar clinical presentation of cecal diverticulitis and acute appendicitis. Future efforts should focus on standardizing the technique, refining patient selection criteria via high-resolution CT imaging, and validating its long-term efficacy against conventional management strategies in larger cohorts.

Despite the promising outcome, this case report has several inherent limitations. First, it describes the experience of a single patient at a single center, which inherently limits the generalizability of our findings. The favorable result may not be replicable in all settings or for all patients. Second, there is a potential for selection bias, as the patient was chosen for this endoscopic approach based on specific anatomical factors (e.g., a single, accessible abscess). The success of this technique is highly dependent on the abscess’s size, location, and communication with the colon, which may not be present in all cases of complicated diverticulitis. Furthermore, the technical expertise required for this procedure may not be readily available in all endoscopic units, potentially limiting its widespread adoption. Finally, we lack long-term follow-up data beyond 1 year to confirm the durability of this treatment and rule out late recurrence. Future prospective studies with larger sample sizes and direct comparisons to standard treatments like percutaneous drainage are warranted to validate these preliminary findings and establish this technique’s precise role in the therapeutic arsenal.

## 4. Conclusion

Endoscopic flushing integrates diagnosis and treatment, and has diagnostic, differential diagnostic, and therapeutic effects on cecum diverticulitis, which provides a new diagnostic and therapeutic idea for the clinical treatment of colonic diverticulitis and is worth further investigation.

## Author contributions

**Data curation:** Chun Gao.

**Funding acquisition:** Chun Gao.

**Investigation:** Chun Gao.

**Methodology:** Jiu-Cong Zhang.

**Project administration:** Jiu-Cong Zhang.

**Supervision:** Chun Gao, Jiu-Cong Zhang.

**Validation:** Chun Gao, Jiu-Cong Zhang.

**Writing – original draft:** Chun Gao.
